# Dynamics of the Reproductive Changes and Acquisition of Oocyte Competence in Nelore (*Bos taurus* *indicus*) Calves during the Early and Intermediate Prepubertal Periods

**DOI:** 10.3390/ani12162137

**Published:** 2022-08-20

**Authors:** Taynan Stonoga Kawamoto, João Henrique Moreira Viana, Thais Preisser Pontelo, Maurício Machaim Franco, Otávio Augusto Costa de Faria, Andrei Antonioni Guedes Fidelis, Luna Nascimento Vargas, Ricardo Alamino Figueiredo

**Affiliations:** 1 Department of Veterinary, Federal University of Uberlandia, Uberlandia 38400-902, MG, Brazil; 2 Animal Reproduction Laboratory, Embrapa Genetic Resources and Biotechnology, Brasília 70770-917, DF, Brazil; 3 Department of Veterinary, Federal University of Lavras, Lavras 37200-900, MG, Brazil; 4 Department of Veterinary, University of Brasilia, Brasília 70910-900, DF, Brazil; 5 Department of Biology, Federal University of Uberlandia, Uberlandia 38400-902, MG, Brazil

**Keywords:** reproductive physiology, rump development, LOPU, oocyte, gene expression, IVEP, embryo

## Abstract

**Simple Summary:**

Brazil has the largest cattle commercial herd in the world, mostly of the Nelore breed (*Bos taurus indicus*), and it is one of the largest in vitro bovine embryo producers. However, Nelore cows experience puberty later than taurine breeds (*Bos taurus taurus*), which can lead to a delay in genetic improvement. Therefore, including calves as oocyte donors for in vitro embryo production programs could be an attractive step for reducing the interval between generations, accelerating the herd’s genetic gains, and contributing to the sustainability of milk and meat production systems. Nevertheless, calves’ oocytes are reported as less competent in generating embryos and establishing pregnancies than those collected from adult females. In addition, most studies have been carried out on taurines. Thus, this study’s goal was to search for a better understanding of prepubertal Nelore females’ reproductive development, related to the performance of their oocytes in terms of in vitro production of embryos. This can provide support for making decisions on the use of this animal category as an oocyte donor and prospects for hormonal protocols or changes in the culture media for in vitro embryo production.

**Abstract:**

The purpose of this study was to characterize the reproductive physiology, oocyte competence, and chromatin compaction in Nelore calves in the early-prepubertal period (EPP) and the intermediate-prepubertal period (IPP). Calves aged 2–5 (EPP) and 8–11 months old (IPP) were assigned to Trial 1 (morpho-physiological–endocrine evaluations, n = 8) or Trial 2 (oocyte donors, n = 8) vs. the respective control groups of cows (n = 8, each). All morphological endpoints, except the antral follicle count, increased from the EPP to the IPP. The EPP LH-FSH plasma concentrations were similar to cows, whereas LH was lower and FSH was higher in the IPP than in cows. . Cows produced more Grade I (12.9% vs. 4.1% and 1.7%) and fewer Grade III COC (30.1% vs. 44.5% and 49.0%) than the EPP and IPP calves, respectively. The IPP calves’ oocyte diameter was similar to those from cows but greater than those from EPP females (124.8 ± 8.5 and 126.0 ± 7.5 μm vs. 121.3 ± 7.5 μm, respectively). The expression of the chromatin compaction-related gene HDAC3 was downregulated in calves. The proportion of the blastocyst rate to the controls was lower in EPP than in IPP calves (43.7% vs. 78.7%, respectively). Progressive oocyte competence was found during the prepubertal period, which can help to decide whether to recover oocytes from calves.

## 1. Introduction

The period from birth until puberty is marked by a number of changes that lead to the maturity of the hypothalamus–hypophysis–ovarian axis and thus prepare the reproductive system for the successful generation of viable offspring. In cattle, as in many other species, the ovary is already active during this period, and follicle recruitment and development will occur up to the antral phase [1,2,3,4]. Sexual steroids produced by growing ovarian follicles promote the development of the tubular part of the reproductive system and play a crucial role in somatic development, acting in fat deposition [5] and in muscle (reviewed by [6]) and bone development [5,7,8]. Ovarian steroids also affect the development of the oocyte.

In vitro embryo production (IVEP) is an artificial reproductive technology that has been widely used in cattle breeding programs worldwide. Over two-thirds of all bovine embryos reported in 2020 were generated by IVEP [9]. The growth of antral follicles during the prepubertal phase enables calves to be used as oocyte donors, and the production of offspring from prepubertal cattle by IVEP has been reported since the 1990s [10]. However, low embryo rates and the technical difficulties of oocyte recovery overcome the economic advantages of using donors with unknown genetic merit. This scenario changed with the recent advances in genomics and the development of commercial microchips to evaluate the genomic predicted transmitting ability (GPTA) for type and production traits in different cattle breeds [11]. Selection of genetically superior cattle can now be determined from birth [11], and this has boosted the demand for the use of prepubertal calves as oocyte donors. This possibility is even more attractive for zebu cattle, which are often less precocious than European breeds. Nelore cattle, the most used *Bos taurus indicus* beef breed in South American countries, have an average age at puberty (around 24 months old [12]) that is almost two-fold the one observed in *Bos taurus taurus* (around 14 months of age [13]).

The use of calves as oocyte donors and thus the generation of offspring before puberty could have a remarkable impact on cattle breeding programs, particularly by accelerating the genetic gain over time. However, embryo rates in prepubertal calves are 10% to 39% [14,15,16,17], contrasting with the 20% to 50% usually obtained from pubertal cows [14,15,18]. These lower rates are observed regardless of the laboratory protocol and have been associated with lower oocyte competence in prepubertal calves and, consequently, lower embryo production [14,15,16,19]. Oocytes recovered from calves present cytoplasmic [20] and/or nuclear [21,22] differences compared with those from adult cyclic cows. Moreover, they are known for having a smaller number of transcripts than those of pubertal bovine females [23]. Since epigenetic factors control gene transcription, one of the possible causes of this difference could be changes in epigenetic factors. Enzymes such as histone acetyltransferase 1 (HAT1), CREB binding protein (CREBBP) and nuclear receptor coactivator 2 (NCOA2) are responsible for histone acetylation, favoring the occurrence of gene transcription. Histone deacetylase 1 (HDAC1), histone deacetylase 2 (HDAC2) and histone deacetylase 3 (HDAC3) are enzymes that are responsible for histone deacetylation, providing an environment in which transcription is unfavorable. Such enzymes are present in bovine oocytes and embryos [24] and appear to be related to the competence of bovine oocytes [25]. 

We hypothesized that oocyte competence could vary throughout the prepubertal period, reflecting the morpho-physiological and endocrine changes observed at different ages. Therefore, the aim of this study was to characterize the reproductive physiology, oocyte competence and chromatin compaction during two time windows of the prepubertal period in Nelore calves. 

## 2. Materials and Methods

### 2.1. Animals and Location

This study was conducted at the Sucupira Experimental Farm and at the Animal Reproduction Laboratory of the Embrapa Genetic Resources and Biotechnology, in Brasilia, Brazil. The contemporaneous Nelore (*Bos taurus indicus*) calves (n = 16) used in the present study were generated by timed artificial insemination (TAI) with X-sorted semen. Non-pregnant, non-lactating, multiparous cows (n = 16) from the same herd were used as controls. Calves were raised on pasture (*Brachiaria decumbens*), with ad libitum access to water and minerals. Weaning occurred at 7 months old (mo). The average daily weight gain during the experiment was 0.57 ± 0.2 kg/day (from an average of 90.9 ± 8.3 kg BW at the beginning to 245.0 ± 12.3 kg BW at the end of the experiment). This study was approved by the Embrapa’s Ethics in the Use of Animals Committee (Protocol CEUA #002/2017).

### 2.2. Experimental Design 

This study aimed to evaluate changes in the morpho-physiological and endocrine characteristics over time, as well as oocyte quality and developmental competence. However, repeated follicle aspiration for cumulus–oocyte complex (COC) recovery is known to alter plasma LH and FSH concentrations [26] and, consequently, to disturb the follicular dynamics [27]. To avoid such bias, calves were randomly allocated into two subsets, which were used in parallel trials to evaluate the physio-endocrine and developmental endpoints (Trial 1, n = 8) or were used as oocyte donors (Trial 2, n = 8). In both trials, two evaluation periods were defined: 2 to 5 (mo), referred as the early prepuberal period (EPP), and 8 to 11 mo, represented by the same prepubertal females in Trial 1 and referred to as the intermediate prepubertal period (IPP). These time windows were chosen to represent the initial and intermediate prepubertal periods, taking into account the expected age at puberty in the Nelore breed (22 to 36 mo, [28]), as well as the ages at which LOPU and OPU are usually performed in calves. Two groups of non-pregnant, non-lactating, multiparous Nelore cows (n = 8 each) were used as the respective controls in Trials 1 and 2.

#### 2.2.1. Trial 1

The experiments for the EPP versus control group (cows) were run from January to April (2018), and those for the IPP group versus the respective controls took place from July to October (2018). In each period, calves were examined weekly by transrectal ultrasonography to measure ovarian and uterine horn diameters (mm), total antral follicle count (AFC), the number of follicles of size ≤ 4 or >4 mm and the diameter of the largest follicle (mm). Blood samples were collected once per day for FSH and twice per day for LH plasma evaluations (at 8:00 am and at 4:00 pm) on the same day as the ultrasonographic exams. Once per month, the calves were weighed and underwent rump biometric measurements. When the calves reached 15 mo, monthly ultrasound scanning was used to determine the age at puberty, based on the first detection of a corpus luteum.

#### 2.2.2. Trial 2

The experiments for the EPP versus control group (cows) were run from March to June (2018), and those for the IPP group versus the respective controls took place from September to December (2018). Calves underwent COC recovery every other week, with a total of six sessions per period. Simultaneously with follicular aspiration of the prepubertal animals, multiparous Nelore cyclic females also had their follicles aspirated (control group). During the EPP, the follicles were aspirated by laparoscopy (LOPU), whereas during the IPP, this was achieved by ultrasound-guided transvaginal follicle aspiration (OPU). The COC recovered in both periods were classified according to their morphological quality, and some (n = 807) were used for IVEP. A subset of the COC (n = 262) was denuded, and the oocytes were measured (diameter) and stored at −80 °C for gene expression analysis. The qPCR was performed with 4 pools of 15 immature oocytes. As a proof of concept, 15 and 14 embryos produced from oocytes recovered from calves at 2 to 5 or at 8 to 11 mo, respectively, were non-surgically transferred to previously synchronized recipients.

### 2.3. Ultrasonographic Evaluations

Ovarian and uterine evaluations were performed using a B-mode ultrasound device (MyLab 30 VetGold, Esaote, Genova, Italy) equipped with a 7.5 MHz linear rectal probe. For all exams, settings related to the frequency, focus depth and gain adjustments were standardized. The AFC and the numbers of follicles of size ≤ 4 or >4 mm were determined visually during ovarian scanning. Linear measurements of the diameter of the ovary, the largest follicle present, and the uterine horns were taken using the internal caliper of the ultrasound. During the first evaluation period (EPP), a probe guide was used to enable transrectal examination without rectal manipulation of the genital tract, whereas for the second period (IPP), as well as for cows, conventional transrectal scanning procedures were used.

### 2.4. Blood Samples and Hormonal Measurements

Blood samples were collected by jugular vein puncture using 21G, double-ended needles and vacuum tubes with EDTA. The samples were kept on ice until being centrifuged (3000× *g* for 15 min), and 1 mL aliquots of plasma were stored at −20 °C. The LH and FSH analyses were performed by radioimmunoassay (RIA), as previously described [29] at the Laboratory of Endocrinology of the Sao Paulo State University (UNESP, Araçatuba, SP, Brazil). The assay sensitivity was 0.126 ng/mL for LH and 0.390 ng/mL for FSH. The intra-assay coefficient of variation was <11%.

### 2.5. Rump Biometric Data

Rump width, length, and geometry were calculated by using the prominences of the tuber ischiae and the tuber coxae of the pelvic bones as reference points. Three-dimensional images of the rump area were acquired by structured infrared light scanning, using a portable sensor (iSense, 3D Systems, Rock Hill, SC, USA) connected to a tablet computer (iPad Air 2, Apple Inc., Cupertino, CA, USA). This computer was equipped with a real-time scanning app (https://itunes.apple.com/us/app/isense/id807510940 (accessed on 10 March 2019)). The equipment’s nominal resolution at 0.5 m distance was 0.9 mm for the x/y axes and 1.0 mm for the z axis (depth). The point cloud data were transformed in a geometric surface and stored as OBJ files. The 3D images were then edited using the open-source software MeshLab (SourceForge, San Diego, CA, USA) to delete unspecific scans from the nearby objects and to perform the measurements. As a validation procedure, manual measurements were taken using a metric tape. There were no significant differences in rump width or length, as measured directly with scales or obtained from the 3D images, and only the latter were used in this study. The rump area was defined as the area of the trapezium formed by the width at the ilium, the width at the ischium and the rump length. Changes in rump geometry were demonstrated in snapshots taken from the back view of 3D images from calves at 2, 5, 8, and 11 mo. The trapeziums were drawn using the same reference points as the biometric measurements.

### 2.6. Oocyte Recovery

During the EPP, oocytes were recovered by LOPU, which required prior sedation of calves. The protocol consisted of an IM injection of 2% xylazine hydrochloride (Sedalex, Venco Saude Animal, Londrina, PR, Brazil), 1% atropine sulfate (Fagra, Sao Paulo, SP, Brazil) and 10% ketamine hydrochloride (Dopalen, Ceva Saude Animal, Paulinea, SP, Brazil). Local anesthesia at the incision sites in the abdominal area was achieved by SC injection of approximately 5 mL 2% lidocaine hydrochloride (Bravet, Rio de Janeiro, RJ, Brazil). A catheter with an insufflator (Inalar compact, NS Group, Sao Paulo, Brazil) connected to its air valve was then introduced, which also served as the access route for the laparoscope. Two more catheters were inserted, one for the anatomical forceps and another for the aspiration system. An aspiration guide connecting a disposable 21G needle to a vacuum pump (59 mmHg negative pressure, WTA Tecnologia, Cravinhos, SP, Brazil) was used. The follicular fluid was collected in conical tubes containing 50 mL Dulbecco’s modified phosphate buffered saline (DPBS), 1% fetal bovine serum (FBS, Life Technologies Co., Grand Island, NY, USA) and 32 mg heparin (Sigma Aldrich, St. Louis, MO, USA) at a temperature of approximately 37 °C. 

During the IPP and in control cows, follicle aspiration was performed with the same device as used for ultrasonographic evaluations of the ovaries but with a micro-convex 7.5 MHz transducer. The OPU procedure used was as previously described [30]. The follicle content recovered was immediately taken to the laboratory for COC identification, classification and IVEP procedures.

### 2.7. Morphology and Oocyte Diameter Analysis

The COC were morphologically classified according to the criteria proposed by Stojkovic et al. [31] as Grade I (homogeneous cytoplasm oocytes and a compact cumulus oophorus with several layers), Grade II (homogeneous cytoplasm oocytes with small irregular pigmentation areas and fewer layers of cumulus cells than in Grade I), Grade III (heterogenous/vacuolated cytoplasm oocytes, fewer than three layers of cumulus or partially denuded cells) or Grade IV (heterogeneous oocytes, and denuded or expanded cumulus cells). The cumulus cells surrounding each oocyte were removed by mechanical pipetting for measuring the oocyte diameter. The diameter (excluding the zona pellucida) considered the mean of two perpendicular measurements of each oocyte. The images were obtained by Motic Images Plus 2.0 software (Motic China Group Co. Ltd., Xiamen, China). 

### 2.8. In Vitro Embryo Production (IVEP)

The IVEP procedures used have been previously described [32]. For in vitro maturation (IVM), 25 to 30 oocytes were placed in 150 μL of maturation media drop. The IVM media consisted of TCM-199 Earl’s salts (Gibco) supplemented with 10% fetal bovine serum (FBS, Gibco), 0.01 IU/mL of follicle-stimulating hormone (FSH), 0.1 mg/mL of L-glutamine, 0.075 mg/mL of amikacin, 0.1 μM of cysteamine and 0.2 mM of sodium pyruvate. The IVM was performed over 22 h at 38.5 °C and 5% CO_2_ in air. After IVM, COCs were transferred to a 150 μL drop of fertilization media, consisting of Tyrode’s albumin lactate pyruvate (TALP), supplemented with 0.5 mM of penicillamine, 0.25 of mM hypotaurine, 25 mM of epinephrine and 10 mg/mL of heparin. The oocytes were co-incubated with the sperm for 18 to 20 h at 38.5 °C and 5% CO_2_ in air. The day of insemination was considered to be Day 0 (D0). Eighteen hours after insemination, the presumptive zygotes of all treatments were washed and transferred to a 150 μL drop of synthetic oviduct fluid media containing amino acids, citrate and inositol (SOFaaci), supplemented with 0.4% bovine serum albumin, and this was incubated at 38.5 °C with 5% CO_2_ for 7 days. Embryos were evaluated on Day 2 (D2) for cleavage and on Days 6 and 7 (D6 and D7) to determine the blastocyst rates.

### 2.9. RNA Extraction, Complementary DNA (cDNA) Synthesis and Real-Time PCR (qPCR)

Total RNA was isolated using the RNeasy Plus MicroKit (Qiagen, Hilden, Germany) according to the manufacturer’s instructions with minor modifications. The total RNA was used for cDNA synthesis using the GoScript Reverse Transcription System (Promega, Madison, WI, USA) according to the manufacturer’s instructions. Reactions were incubated at 70 °C for 5 min, 42 °C for 60 min and 70 °C for 15 min.

Real-time quantitative polymerase chain reactions (RT-qPCRs) were performed using the Fast SYBR Green Master Mix kit (Applied Biosystems). Each sample was analyzed in triplicate, and PCR specificity was determined by examining the melting curves and amplicon sizes on an agarose gel. Reactions were performed in a final volume of 25 μL using template cDNA equivalent to 0.6 of an oocyte. The PCR conditions were 95 °C for 5 min followed by 50 cycles of denaturation at 95 °C for 10 s, and then annealing and extension at 60 °C for 30 s. The names, primer sequences, amplicon sizes and annealing temperatures of the genes analyzed are listed in Table 1. GAPDH and β-ACTIN were taken as the endogenous control genes. The relative expression of each gene was calculated using the ΔΔCt method with efficiency correction by the Pfaffl method [33].

### 2.10. Statistical Analysis

The data of continuous variables were first examined for normality and homogeneity of variance using the Shapiro–Wilk and Bartlett tests. Variables with a normal distribution were evaluated by ANOVA, and differences between animal categories (calves vs. cows) or between prepubertal periods (EPP vs. IPP) were compared using the Student’s t test. Otherwise, the data were analyzed using the Mann–Whitney or Kruskal–Wallis non-parametric tests. In order to compare embryos rates between EPP and IPP, the data were transformed as proportions of their respective control groups (cows). The associations between reproductive and biometric data were evaluated using Spearman’s correlation. The reproductive developmental rate was evaluated by non-linear regression analysis (Gompertz curve), and the validation of this curve was tested by the deviance of the fitted curve compared with a straight line. The Gompertz model equation was Z*exp(−b*exp(a*x)), where Z is the maximum data value, b is the integration constant and a corresponds to the acceleration rate. This non-linear regression analysis (Gompertz curve) was also used to compare changes in rump geometry at different ages. All statistical procedures were performed using GraphPad Prism (v. 6) or R (v. 3.6.1) software, and a *p* value of ≤0.05 was considered statistically significant. The results are shown as means ± SEM.

## 3. Results

### 3.1. Trial 1

**Figure 1:**
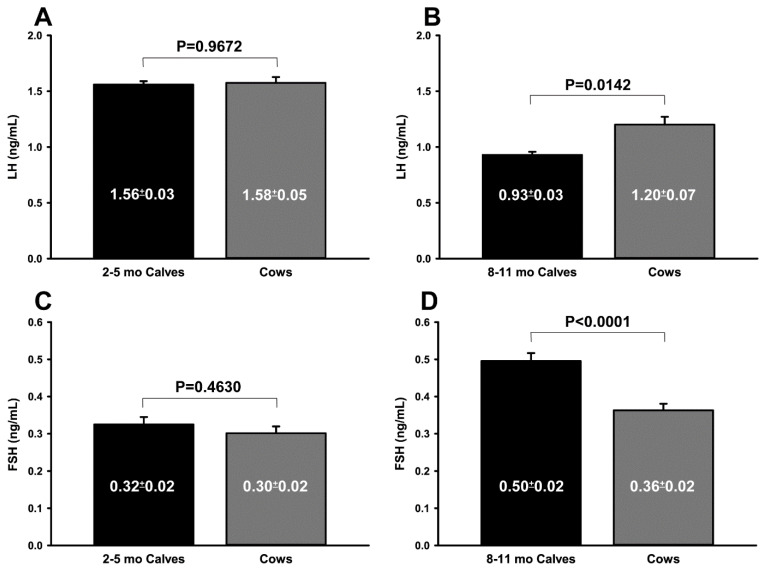
(**A**–**D**) Comparison of plasma LH (**A**,**B**) and FSH (**C**,**D**) concentrations (ng/mL) in Nelore (*B. taurus indicus*) calves during two prepubertal periods (black bars) and multiparous Nelore cows (gray bars). (**A**) LH, 2 to 5 mo calves vs. cows; (**B**) LH, 8 to 11 mo calves vs. cows; (**C**) FSH, 2 to 5 mo calves vs. cows; (**D**) FSH, 8 to 11 mo calves vs. cows. *p* values were determined using the t test (**A**,**B**) or the Mann–Whitney test (**C**,**D**). The results are shown as means ± SEM.

When the ultrasonographic evaluation data of prepubertal ovaries and the uterus were compared with those in cows, most parameters of the control groups were higher than those of the EPP or IPP groups (Table 2 and Table 3). The EPP vs. IPP ultrasonographic data are described in Table 4. The ovarian and the uterine diameter, the number of follicles larger than 4 mm, and the diameter of the largest follicle were greater in calves during the IPP than during the EPP (*p* < 0.001). However, there was no difference (*p* > 0.05) in AFC between the EPP and IPP. During the EPP, calves had LH and FSH plasma concentrations similar (*p* > 0.05) to those of cows, whereas during the IPP, calves had lower LH (*p* < 0.05) and higher FSH (*p* < 0.001) concentrations than cows (Figure 1A–D).

**Figure 2:**
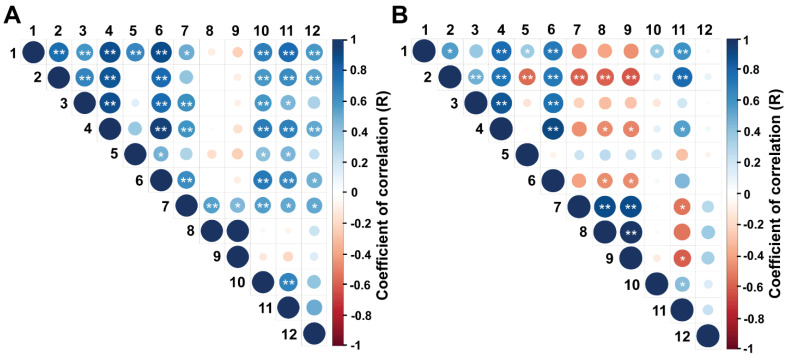
(**A**,**B**) Spearman’s correlation between reproductive and biometric parameters in Nelore (*B. taurus indicus*) calves at (**A**) 2 to 5 mo and (**B**) 8 to 11 mo. The endpoints evaluated were: 1, rump width at the ilium (cm); 2, rump width at the ischium (cm); 3, rump length (cm); 4, rump area (cm^2^); 5, ratio between the lengths of the ilium and ischium; 6, body weight (kg); 7, ovarian diameter (mm); 8, antral follicle count; 9, number of follicles ≤ 4.0 mm; 10, number of follicles > 4.0 mm; 11, diameter of the largest follicle (mm); 12, uterine horn diameter (mm). * *p* < 0.05 and ** *p* < 0.01.

The correlation matrix for the reproductive and biometric endpoints is shown in Figure 2A,B. During the EPP, moderate to high positive correlations were observed among ovarian diameter, number of follicles of size > 4.0 mm, diameter of the largest follicle, uterine horn diameter and rump biometric endpoints (R values ranging from 0.30 to 0.77, *p* < 0.05). Conversely, during the IPP, most of these associations were weak and non-significant (*p* > 0.05). In both periods, AFC and the number of follicles ≤ 4 mm only presented a significant positive correlation with ovarian diameter, whereas in the IPP, AFC and the number of follicles of size ≤ 4 mm were negatively correlated with rump width at the ischium, rump area and body weight (R = −0.10, −0.16, and −0.10, respectively; *p* < 0.05).

**Figure 3:**
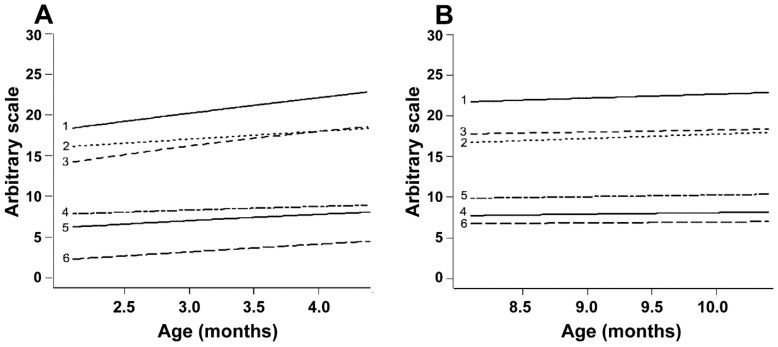
(**A**,**B**) Gompertz curves for reproductive parameters in Nelore (*B. taurus indicus*) calves from 2 to 5 mo (**A**) and from 8 to 11 mo (**B**). The parameters evaluated and their respective r^2^ values in Periods A and B are, respectively, as follows: 1, antral follicle count (r^2^ = 0.056 and 0.001); 2, follicles ≤ 4.0 mm (r^2^ = 0.014 and 0.002); 3, ovarian diameter (r^2^ = 0.442 and 0.009); 4, diameter of the largest follicle (r^2^ = 0.207 and 0.014); 5, uterine horn diameter (r^2^ = 0.157 and 0.024); 6, ovarian follicles > 4.0 mm (r^2^ = 0.232 and 0.001). The R software output presented the mean values on the Y axis with an arbitrary scale so that all endpoints fitted in the same picture.

The Gompertz model was used to analyze whether the differences observed between correlations in the EPP and IPP were caused by differences in the rate of reproductive development (Figure 3A). During the EPP, the ovarian diameter and the number of follicles of size > 4.0 mm increased by 33%, the diameter of the largest follicle increased by 24% and the uterine horn diameter increased by 19%. To ensure that the slope of the curves was positive, they were compared with a straight line. The curves for all endpoints, except follicles of size ≤ 4.0 mm, differed (*p* < 0.05) from the straight line, i.e., they represented growing trends (Table 5). Conversely, during the IPP, the equations for the same endpoints showed r^2^ values below 0.01 (Figure 3B), and the curves did not differ from straight lines (*p* > 0.05, Table 5). The following equations were calculated for the EPP:

Ovarian diameter: 23.57 ∗ exp(−1.00983 ∗ exp(−0.33040 ∗ x)); r^2^ = 0.44

Follicles > 4.0 mm: 8 ∗ exp(−2.52892 ∗ exp(−0.33549 ∗ x)); r^2^ = 0.23

Diameter of the largest follicle: 11.30 ∗ exp(−0.99893 ∗ exp(−0.24732 ∗ x)); r^2^ = 0.21

Uterine horn diameter: 11.20 ∗ exp(−0.53403 ∗ exp(−0.19523 ∗ x)); r^2^ = 0.16

Rump area: 711.40 ∗ exp(−2.05001 ∗ exp(−0.64280 ∗ x)); r^2^ = 0.748

The following equations were calculated for the IPP:

Ovarian diameter: 23.65 ∗ exp(−0.43306 ∗ exp(−0.05201 ∗ x)); r^2^ = 0.009

Follicles > 4.0 mm: 14 ∗ exp(−0.84750 ∗ exp(−0.02280 ∗ x)); r^2^ = 0.002

Diameter of the largest follicle: 10.65 ∗ exp(−0.62063 ∗ exp(−0.08197 ∗ x)); r^2^ = 0.014

Uterine horn diameter: 13.15 ∗ exp(−0.55009 ∗ exp(−0.08051 ∗ x)); r^2^ = 0.024

Rump area: 1026.22 ∗ exp(−3.7862 * exp(−0.3416 ∗ x)); r^2^ = 0.303

**Figure 4:**
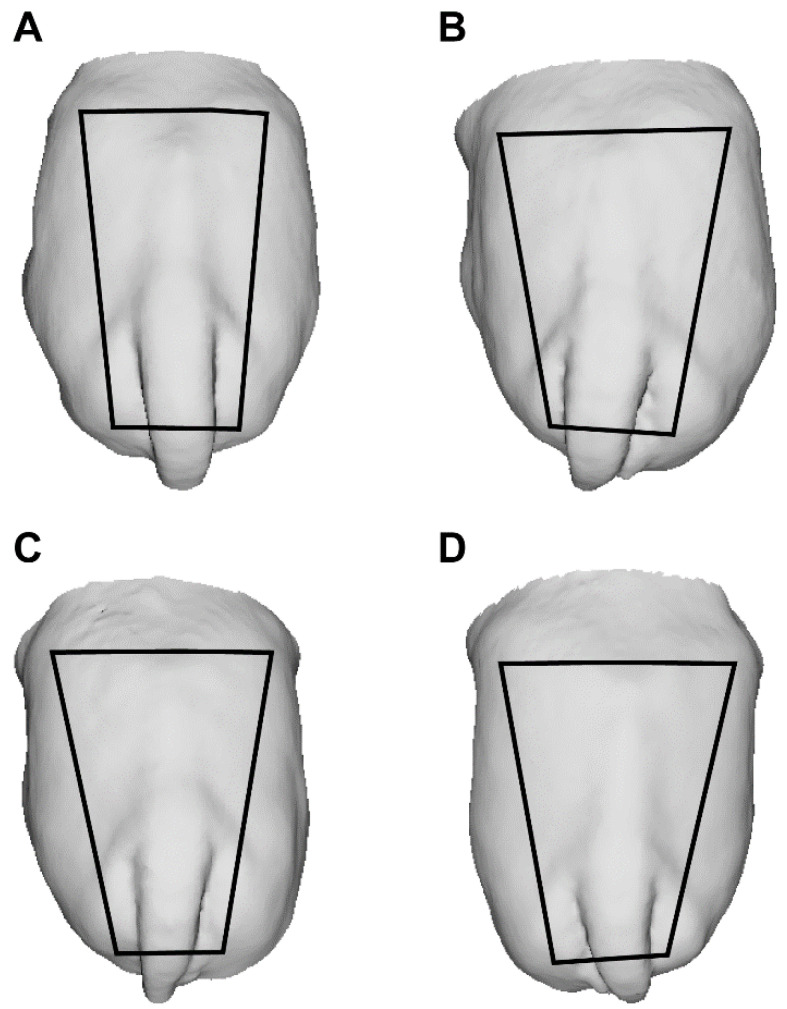
(**A**–**D**) Changes in rump geometry during prepubertal development in Nelore (*B. taurus indicus*) calves. Back-view 3D images of Nelore females’ rumps at 2 (**A**), 5 (**B**), 8 (**C**) and 11 (**D**) mo. Trapezoids show changes in the rump geometry. To build the trapeze, the iliac crest and tuber ischiadicum were used as reference points.

**Figure 5:**
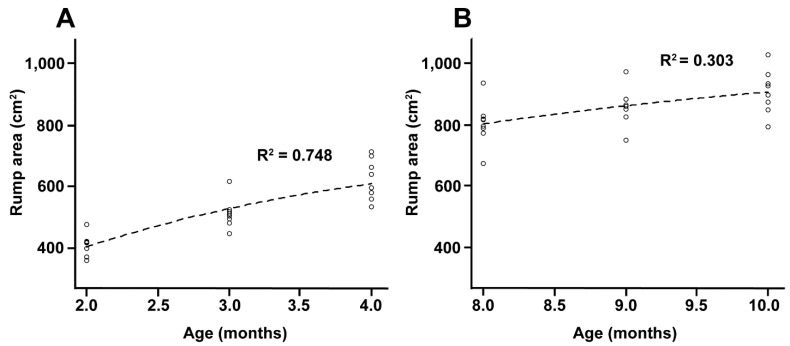
(**A**,**B**) Non-linear regression curves of the r^2^ values of the rump geometry measurements in Nelore (*B. taurus indicus*) calves at 2 to 5 mo (**A**) and 8 to 11 mo (**B**).

Changes in rump geometry are shown in Figure 4A–D. The non-linear regression analysis demonstrated a greater developmental rate in rump area during the EPP, as it increased by 64% (r^2^ = 0.75) compared with 34% (r^2^ = 0.31) during the IPP (Figure 5A,B). During the EPP, there was also a greater change in rump geometry, which changed from a square to a trapezoidal shape (Figure 4A,B). The average rump area was 1684.2 ± 173.8 cm^2^ for cyclic cows (control) and 514.2 ± 101.4 and 856.72 ± 79.1 cm^2^ for EPP and IPP calves, respectively. The ratio of rump width at the ilium to that at the ischium differed between the EPP and IPP groups (*p* < 0.05). The calves reached puberty at an average of 20.5 mo (ranging from 17 to 24 mo).

### 3.2. Trial 2

In total, 1817 oocytes were recovered, of which 362 oocytes were measured for size. The IPP calves had an average oocyte diameter similar to those of the control cows (124.8 ± 8.5 vs. 126.0 ± 7.5 μm, respectively; *p* > 0.05) but greater than those of EPP calves (124.8 ± 8.5 vs. 121.3 ± 7.5 μm, respectively; *p* = 0.012). Regarding morphological quality, the control cows had a greater percentage of Grade I oocytes compared with calves, regardless of the prepubertal period (Table 6).

The blastocyst rate on D7 was greater in control cows than in EPP calves (71.6% vs. 31.0%, respectively; *p* = 0.0001; Table 7) but not in IPP calves (48.1% vs. 42.0%; *p* = 0.22; Table 8). A direct comparison between blastocyst rates during the EPP and the IPP was not possible, as IVEP was performed at different moments. When the data were transformed to the relative proportion of the control group (cows), however, embryo production was greater (*p* < 0.05) during the IPP than during the EPP, as shown in Table 9.

**Figure 6:**
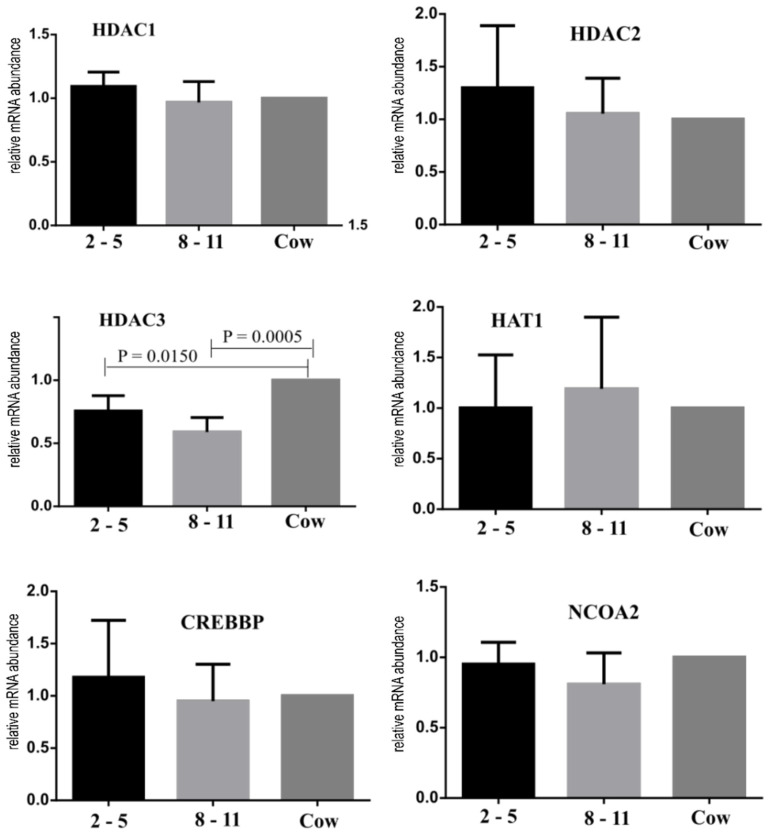
Gene expression of HDAC1, HDAC2, HDAC3, HAT1, CREBBP and NCOA2 in immature oocytes of Nelore (*B. taurus indicus* 2 to 5 mo calves (black bar), 8 to 11 mo calves (light gray bar), and control cows (dark gray bar)). The p value is given in the figure when a statistically significant difference (*p* < 0.05) was found.

HDAC3 was downregulated (*p* < 0.05) in immature oocytes recovered from calves when compared with those from control animals, whereas no difference (*p* > 0.05) was found for the expression of the HAT1, HDAC1, HDAC2, CREBBP and NCOA2 genes (Figure 6). 

A subset of the embryos produced from calves (n = 29) was transferred to previously synchronized recipient cows. From the 15 embryos originating during the EPP, five generated pregnancies at 23 days post-transfer, but one was lost at 60 days, resulting in four offspring. Conversely, from the 14 embryos originating during the IPP, two generated pregnancies 23 days post-transfer, of which one was lost after 60 days, resulting in one offspring.

## 4. Discussion

The aim of this study was to characterize the changes in reproductive, biometric and endocrine patterns, as well as oocyte developmental potential during the early (EPP) and intermediate (IPP) prepubertal periods in Nelore (*B. taurus indicus*) calves. In this regard, we ran parallel trials with two groups of contemporaneous calves in order to avoid bias due to the potential interference of OPU on the endocrine and physiologic ovarian endpoints. The present results support the hypothesis that the dynamics of these changes during the prepubertal period differ over time and are associated with a progressive gain in developmental competence by the oocytes. 

There was a progressive increase in ovarian activity during the prepubertal period, demonstrated by the difference in the number of growing follicles and in the maximum size of the largest (presumed to be dominant) follicle, and indirectly by the uterine horn diameter between the EPP (2 to 5 mo) and IPP (8 to 11 mo) calves. However, the dynamics of ovarian and uterine development differed between periods, with a higher growth rate being observed during the EPP, as demonstrated by the differences in the non-linear Gompertz curves. In EPP calves, there was a significant increase in all endpoints other than AFC, whereas in IPP calves, the development curves were not significantly different from a straight line, i.e., relative stability. The faster development observed in younger calves is in line with the so-called “mini puberty” previously described in *B. taurus* [1] as well as in sheep [4] and humans (reviewed by [3]). Coherently, ovarian activity (i.e., follicular growth) showed positive and significant correlations with ovarian and uterine horn diameters, as well as with biometric endpoints (length at ilium, length at ischium, rump length, rump area, ratio between rump width at ilium to that ischium and body weight) during the EPP. However, most of these correlations became non-existent or even negative in IPP calves.

Changes in rump geometry (from squared to trapezoid) were observed only in EPP calves, and further development was characterized mainly by increases in rump size rather than by changes in shape. Rump characteristics are associated with pregnancy failure, anestrus and dystocia [34,35,36] in heifers, highlighting the importance of the changes in rump development that take place during the early prepubertal phase. A novelty of the current study was the use of 3D image technologies to demonstrate these changes in rump geometry. Structured light scanning allows for the fast and accurate generation of 3D models from cattle, which can be freely rotated in a 3D virtual space and used to define reference marks and acquire biometric measurements [37]. In the current study, rump size and geometry were calculated using the prominences of the tuber ischiae and the tuber coxae of the pelvic bones as references, which can be easily spotted in the 3D models.

Unlike the other ovarian endpoints evaluated, the number of ultrasonographically detectable antral follicles (AFC) did not differ between age groups, and analysis by the Gompertz curve showed little or no trend toward an increase in AFC values within each period analyzed. Coherently, in both periods, AFC was positively correlated only with ovarian diameter and, in older calves, negatively correlated with rump development and body weight. The association between AFC and ovary size has previously been described in pubertal cattle [38]. In the current study, we showed that this relationship also occurs in early prepubertal calves. As AFC presents high individual repeatability from prepubertal to post-pubertal heifers [39], our findings suggest that oocyte donor selection can be performed in young calves, independent of other characteristics associated with prepubertal sexual development. However, possible interplay between AFC and somatic development remains to be investigated.

The progressive increase in the number of growing follicles and in the maximum size of the largest follicle during the EPP, as observed in this study, is consistent with the hypothesis that a greater hypophyseal release of FSH and LH occurs during early calfhood. Plasma concentrations of FSH and LH in EPP calves were similar to those in the control cows. The hypophyseal gonadotropins support follicle growth and steroidogenesis (reviewed by [40]), which, in turn, may promote uterine development and changes in rump geometry, as observed in the current study. Mauras et al. [41] observed that in children, an increase in the GnRH pulse amplitude leads to greater FSH and LH release and subsequent estradiol production, which, in turn, increases the production of growth hormone (GH) and insulin-like growth hormone (IGF-1), as well as calcium absorption and skeleton mineralization.

Conversely, the events observed during the IPP support the hypothesis of the increased sensitivity of the hypothalamic–hypophyseal axis in response to the negative feedback from estradiol. Previous studies have shown that after the transient hypothalamic–pituitary–gonadal axis activation soon after birth, a decrease in the plasma concentration of gonadotropins takes place [1,4]. In our study, LH concentrations were lower in IPP calves than in control cows. The lower amplitude and frequency of LH pulses may have reduced estradiol production, which could explain the lower rate of development of primary and secondary sexual characteristics during this period. Conversely, the consequent lower negative feedback on FSH production [42] would account for the plasma concentrations of FSH observed in IPP calves, which were higher than those from control cows. 

Remarkable differences were also observed between the EPP and the IPP groups regarding oocyte quality. Although the control cows yielded more Grade I COC than calves, regardless of the age, oocytes recovered during the IPP, but not during the EPP, and had a similar diameter and developmental potential, compared with those from adult cows. Follicle growth during the EPP occurred under LH and FSH concentrations similar to those observed in adult cattle, i.e., gonadotrophin stimulation alone does not explain the lower oocyte developmental potential observed in this phase. A number of protocols based on exogenous FSH or eCG stimuli have been proposed to increase follicle size at aspiration and thus blastocyst rates in 2 to 6 mo prepubertal donors, but the results are generally lower than those observed in pubertal cattle, albeit better than in non-stimulated calves [16,17]. One could speculate that the LH and FSH release pattern, rather than the average circulating concentrations, may be the key to controlling the acquisition of competence by the oocyte. Conversely, it is also possible that the higher FSH concentrations during the IPP, which supported follicle growth to greater diameters, may have promoted a better intrafollicular microenvironment and the proper accumulation of organelles and messenger RNA needed to support further embryo development [20]. 

The blastocyst rates reported in the literature for oocytes recovered from prepubertal cattle are quite varied, ranging from 0% to 39% [14,15,16,17,19,21] in animals submitted or not submitted to hormonal pretreatment. Our results highlight the importance of considering the age of the calves collected in each study, as oocyte competence changes throughout the prepubertal period. In the current study, the developmental potential of oocytes from 8 to 11 mo calves was already similar to those from cows, regardless of the fact that these same calves only reached puberty at 20.5 mo on average, i.e., more than six months later. In this regard, the mechanisms limiting the onset of puberty in the late prepubertal period (>12 months) in the Nelore breed do not seem to be connected to oocyte competence. 

Comparisons of the blastocyst rates among studies should also consider differences in the IVEP protocols, media, and staff skill among laboratories, as well as the natural fluctuation of environmental effects over time. In the current study, to avoid bias due to differences in genetic background, we evaluated the blastocyst rates of the same calves but during two time windows (EPP and IPP). The numeric values obtained (31.0 and 42.0%, respectively) were only slightly different. However, when the data were transformed to the proportion of the blastocyst rates to their respective control (adult) groups, differences in developmental potential became evident (43.7% vs. 78.8% for 2 to 5 and 8 to 11 mo calves, respectively).

Some studies have suggested a possible association between histone acetylation levels and the acquisition of oocyte competence [43,44,45]. In the present study, we investigated potential differences in chromatin compaction of oocytes from calves. A higher amount of HDAC3 transcript was observed in cows than in calves. The HAT1, CREBBP and NCOA2 genes are involved in histone acetylation, and the HDAC1, HDAC2 and HDAC3 genes are involved in histone deacetylation. Histone acetylation and deacetylation control chromatin opening and thus higher transcriptional activity, playing a key role in the preparation of oocytes for maturation and further resumption of meiosis [46]. Thus, the higher amount of HDAC3 transcripts observed in cows in this study may suggest that these oocytes would be better prepared for the chromatin condensation that occurs during the first 8 h of in vitro maturation. Changes in the chromatin configuration along with the epigenetic events at the beginning of meiotic maturation would promote gene silencing, occurring in parallel with the acquisition of oocyte competence [47,48,49]. Oocytes from prepubertal cattle are known to show fewer total transcripts than those from pubertal ones [23,50,51]. Thus, one of the possible causes for the lower number of transcripts observed in prepubertal oocytes could be due to down- or upregulation of epigenetic factors. Despite all the differences found between the prepubertal oocytes and those of cows, in this proof of concept, the embryos were able to generate some pregnancies, which had full development and some healthy offspring. This was also reported in other studies [16,52,53], showing the reproduction technique’s feasibility, including the use of prepubertal animals as oocyte donors.

## 5. Conclusions

In summary, the EPP in Nelore calves is characterized by increased ovarian activity, uterine development and rump geometry changes, contrasting with the relative stability observed in the IPP. The transition from the EPP to the IPP is characterized by a progressive acquisition of oocyte developmental competence, which in 8 to 11 mo calves, is similar to that in cows. These findings highlight the importance of the early prepubertal period for the development of primary and secondary characteristics, and they shed light on the implications of the differences in physiology between these periods for the use of calves as oocyte donors.

## Figures and Tables

**Table 1:** Sequence of optimized primer oligonucleotides, amplified fragment size in bp (base pairs) and annealing temperature.

Gene	Sequence	Fragment Size (pb)	Annealing Temperature (°C)	GenBank
GAPDH	F: GGC GTG AAC CAC GAG AAG TAT AA	119	60	NM_001034034.2
	R: 5′ CCC TCC ACG ATG CCA AAG T 3′			
ACTB	F: GGC ACC CAG CAC AAT GAA GAT CAA	134	60	NM_173979.3
	R: 5′ ATC GTA CTC CTG CTT GCT GAT CCA 3			
CREBBP	F: GTT CTC CAC TAC GAC ATC ATC	150	60	NM_001164022.1
	R: CTT GTT GAC TCG GTC TTC C			
NCOA2	F: CCT GGG ATG GAC ATG ATT AAG	125	60	XM_027561151.1
	R: TGG GTC GAA ACG AAG AGA			
HAT1	F: AAT TGA GAG ACT TTG TGC TTG TGA	392	60	NM_001034347.1
	R: TTC AAT GAC ACG TCG ATA ATC TTC			
HDAC1	F: ATC GGT TAG GTT GCT TCA ATC TG	188	60	NM_001037444.2
	R: GTT GTA TGG AAG CTC ATT AGG GA			
HDAC3	F: GAA GAG GCC ATT AGT GAA GAG	227	60	NM_001206243.1
	R: TCA GTC CTG TCG TAG GTT AG			
HDAC2	F: TTC CTG GAA CAG GAG ACT TA	194	60	NM_001075146.1
	R: ATC ACC AGA TAG GGA GTC TG			

**Table 2:** Average values (mean ± SD) of reproductive and somatic endpoints in Nelore cattle (*B. taurus indicus)* in two animal categories: 2 to 5 months old and cows.

Endpoint	Animal Category		*p* Value
	2 to 5 months	Cow	
Ovarian diameter (mm)	16.5 ± 2.4	25.1 ± 2.4	<0.0001
Antral follicle count (n)	20.7 ± 6.7	28.3 ± 7.4	<0.0001
Number of follicles > 4 mm	3.4 ± 1.6	6.0 ± 3.8	<0.0001
Diameter of the largest follicle (mm)	7.2 ± 1.5	10.1 ± 2.6	<0.0001
Uterine diameter (mm)	8.4 ± 1.0	12.8 ± 1.8	<0.0001
Mean rump area (cm^2^)	514.2 ± 101.4	1684.2 ± 185.8	<0.0001

*t* test and Mann–Whitney analysis, *p* < 0.05.

**Table 3:** Average values (mean ± SD) of reproductive and somatic endpoints in Nelore cattle (*B. taurus indicus)* in two animal categories: 8 to 11 months old and cows.

Endpoint	Animal Category		*p* Value
	8 to 11 months	Cow	
Ovarian diameter (mm)	18.1 ± 2.1	24.1 ± 2.6	<0.0001
Antral follicle count (n)	22.0 ± 8.9	26.4 ± 7.7	0.0001
Number of follicles > 4 mm	4.9 ± 2.2	4.8 ± 2.2	0.9583
Diameter of the largest follicle (mm)	7.9 ± 1.3	9.6 ± 2.3	<0.0001
Uterine diameter (mm)	10.1 ± 1.0	12.5 ± 1.3	<0.0001
Mean rump area (cm^2^)	856.72 ± 79.1	1684.2 ± 185.8	<0.0001

*t* test and Mann–Whitney analysis, *p* < 0.05.

**Table 4:** Average values (mean ± SD) of reproductive and somatic endpoints in Nelore (*B. taurus indicus*) calves during two time-windows in the prepubertal period: from 2 to 5 and from 8 to 11 months old.

Endpoint	Prepubertal Period		*p* Value
	2 to 5 months	8 to 11 months	
Ovarian diameter (mm)	16.5 ± 2.4	18.1 ± 2.1	<0.0001
Antral follicle count (n)	20.7 ± 6.7	22.0 ± 8.9	0.5412
Number of follicles > 4 mm	3.4 ± 1.6	4.9 ± 2.2	<0.0001
Diameter of the largest follicle (mm)	7.2 ± 1.5	7.9 ± 1.3	0.0001
Uterine diameter (mm)	8.4 ± 1.0	10.1 ± 1.0	<0.0001
Average body weight	118.8 ± 22.9	227.0 ± 18.7	<0.0001
Mean rump area (cm^2^)	619.5 ± 131.5	967.0 ± 56.25	<0.0001

*t* test and Mann–Whitney analysis, *p* < 0.05.

**Table 5:** Comparison between the Gompertz curves for reproductive parameters (as explained in Figure 3) and a straight line in 2 to 5 and 8 to 11 mo Nelore (*B. taurus indicus*) calves. Results are shown as the sum of squares (SQ), F, and *p* values. When *p* < 0.05, the curve’s slope was considered to be positive.

Endpoint	Age Group					
	2 to 5 months			8 to 11 months		
	SQ	F	*p*-Value	SQ	F	*p* Value
Ovary diameter (mm)	−238.9	74.6	<0.001	−4.3	0.9	0.3
AFC (n)	−244.0	5.6	0.02	−16.3	0.1	0.6
Follicles ≤ 4.0 mm (n)	−61.1	1.3	0.26	−19.8	0.2	0.6
Follicles > 4.0 mm (n)	−60.1	28.4	<0.001	−0.9	0.2	0.6
Largest follicle (mm)	−42.3	24.5	<0.001	−2.4	1.5	0.2
Uterine diameter (mm)	−15.2	17.5	<0.001	−2.9	2.6	0.1

**Table 6:** Oocyte diameter (μm) and oocyte morphological quality grade (%) of control cows or prepubertal calves at 2 to 5 or 8 to 11 mo in the Nelore (*B. taurus indicus*) breed.

Group	Oocyte Diameter (mean ± SD, n)	Oocyte Quality Grade (n, %)			
		GI	GII	GIII	GIV
Calves, 2 to 5 mo	121.3 ± 7.5 ^b^ (85)	9 (1.7) ^b^	134 (25.0) ^a^	262 (49.0) ^a^	130 (24.3) ^b^
Calves, 8 to 11 mo	124.8 ± 8.5 ^a^ (78)	16 (4.1) ^b^	49 (12.6) ^b^	173 (44.5) ^a^	151 (38.9) ^a^
Control cows	126.0 ± 7.5 ^a^ (99)	115 (12.9) ^a^	279 (31.2) ^a^	269 (30.1) ^b^	230 (25.7) ^ab^

^a,b^ Values in the same column followed by different superscripts (*p* < 0.05).

**Table 7:** Cleavage and blastocyst rates on Days 6 (D6) and 7 (D7) obtained with oocytes collected from control cows or 2 to 5 mo Nelore (*B. taurus indicus*) calves.

Group	N	Cleaved n (%)	Blastocyst D6 n (%)	Blastocyst D7 n (%)
Control cows	247	214 (86.6)	122 (49.4) ^a^	177 (71.6) ^a^
Calves, 2 to 5 mo	171	121 (70.8)	27 (15.8) ^b^	53 (31.0) ^b^

^a,b^ Values in the same column followed by different superscripts (*p* < 0.05).

**Table 8:** Cleavage and blastocyst rates on Day 6 (D6) and Day 7 (D7) obtained with oocytes collected from control cows or 8 to 11 mo Nelore (*B. taurus indicus*) calves.

Group	N	Cleaved n (%)	Blastocyst D6 n (%)	Blastocyst D7 n (%)
Control cows	270	193 (71.5)	90 (33.3)	130 (48.1)
Calves, 8 to 11 mo	119	89 (74.8)	22 (18.5)	50 (42.0)

**Table 9:** Blastocyst rates after IVEP using oocytes collected from Nelore (*B. taurus indicus*) calves in two time windows of the prepubertal period, presented as a proportion of their respective cow controls.

Category	D6/Control (%)	D7/Control (%)
2 to 5 mo	31.04	43.78 ^a^
8 to 11 mo	59.63	78.77 ^b^

^a,b^ Values in the same column followed by different superscripts (*p* < 0.05).

## Data Availability

Not applicable.
